# Is Extra Virgin Olive Oil an Ally for Women's and Men's Cardiovascular Health?

**DOI:** 10.1155/2020/6719301

**Published:** 2020-04-24

**Authors:** Flavia Franconi, Ilaria Campesi, Annalisa Romani

**Affiliations:** ^1^Laboratorio Nazionale sulla Farmacologia e Medicina di Genere, Istituto Nazionale Biostrutture Biosistemi, 07100 Sassari, Italy; ^2^Dipartimento di Scienze Biomediche, Università Degli Studi di Sassari, 07100 Sassari, Italy; ^3^Laboratorio PHYTOLAB (Pharmaceutical, Cosmetic, Food Supplement Technology and Analysis), DiSIA Università Degli Studi di Firenze, 50019 Florence, Italy; ^4^Laboratorio di Qualità Delle Merci e Affidabilità di Prodotto, Università Degli Studi di Firenze, 59100 Florence, Italy

## Abstract

Noncommunicable diseases are long-lasting and slowly progressive and are the leading causes of death and disability. They include cardiovascular diseases (CVD) and diabetes mellitus (DM) that are rising worldwide, with CVD being the leading cause of death in developed countries. Thus, there is a need to find new preventive and therapeutic approaches. Polyphenols seem to have cardioprotective properties; among them, polyphenols and/or minor polar compounds of extra virgin olive oil (EVOO) are attracting special interest. In consideration of numerous sex differences present in CVD and DM, in this narrative review, we applied “gender glasses.” Globally, it emerges that olive oil and its derivatives exert some anti-inflammatory and antioxidant effects, modulate glucose metabolism, and ameliorate endothelial dysfunction. However, as in prescription drugs, also in this case there is an important gender bias because the majority of the preclinical studies are performed on male animals, and the sex of donors of cells is not often known; thus a sex/gender bias characterizes preclinical research. There are numerous clinical studies that seem to suggest the benefits of EVOO and its derivatives in CVD; however, these studies have numerous limitations, presenting also a considerable heterogeneity across the interventions. Among limitations, one of the most relevant in the era of personalized medicine, is the non-attention versus women that are few and, also when they are enrolled, sex analysis is lacking. Therefore, in our opinion, it is time to perform more long, extensive and lessheterogeneous trials enrolling both women and men.

## 1. Introduction

The Mediterranean diet (MedDiet) includes high consumption of legumes, cereals, fruits, and vegetables; moderate fish and wine consumption; and low consumption of red meat ([1] and cited literature). The MedDiet also includes the consumption of 25–50 ml/day of extra virgin olive oil (EVOO), which seems to have health benefits [2, 3].

Cardiovascular diseases (CVD) are the main cause of deaths, accounting for >17 million deaths annually [4]. The beneficial effect of MedDiet on CVD is suggested by several randomized clinical trials, although some recent papers stated that the evidence is still uncertain [5, 6]. For example, the Oslo Diet-Heart Study and the Finnish Mental Hospital Study [7–9] tested the effectiveness of low-cholesterol diets, enriched in polyunsaturated fatty acids, showing a decrease in coronary heart diseases (CAD) and blood cholesterol (Chol). Moreover, the Seven Countries Study, enrolling 11,579 middle-aged men from eight nations of seven Mediterranean and non-Mediterranean countries, shows a lower mortality from ischemic heart disease (IHD) in Mediterranean populations compared to those of Northern Europe and America [10]. PREDIMED study proves that EVOO is linked to lower risk of cardiovascular (CV) events [11]. However, a Cochrane Systematic Review proves that elevation in polyunsaturated fatty acids (PUFA) assumption has a small effect, if any, on all-cause mortality or CV deaths although it slightly decreases Chol and probably triglycerides (TG), leaving practically unaltered high density lipoprotein (HDL) [12].

Beneficial effects of EVOO are also associated with the presence of minor polar compounds (MPCs) that have antioxidant, anti-inflammatory, anti-aggregating, and antimicrobial activities and regulate serum insulin/glucose response [13–21]. A claim of the European Food Safety Authority (EFSA) declared that “consumption of olive oil polyphenols contributes to the protection of blood lipids from oxidative damage” at a daily dose of 5 mg of hydroxytyrosol (HTyr) and its derivatives (e.g., oleuropein complex and tyrosol) [22].

Actually, botanicals are largely used [23, 24], especially by women [25, 26], but rigorous findings regarding their efficacy and safety profiles are still lacking [27]. Besides, the influence of sex on botanicals including EVOO, VOO, OO, and MPCs is also lacking; nevertheless, the individual's sex and gender is one of the most important modulators of CV health [28–39] and the numerous sex and gender differences at CV level are summarized in Table 1. Previously, we reviewed the sex-gender effect on polyphenols of various origins [25, 26]; here we focus on EVOO and its MPCs because, as already mentioned, EFSA declares their utility in ameliorating low-density lipoproteins (LDL) oxidation and their importance in MedDiet [22].

## 2. MedDiet and Sex Differences

The Mediterranean Region includes about 20 nations with different ethnic, historical, and cultural backgrounds; religions (Muslims, Orthodox Christians, Catholic Christians, Jews); and economic status [56], and the UNESCO declared that MedDiet is an intangible cultural heritage [57]. Importantly, MedDiet also includes social aspects (social integration) and a peculiar way of life (sleeping and nutrition) that may play a role in reducing age-related diseases [58, 59]. However, the transferability of the benefit of MedDiet outside of Mediterranean Region decreases the importance of social aspects [60, 61]. In particular, it has been found that, in US women who are adherent to MedDiet, the CV risk reduced by about 25% over 12 years, having a reduction in myocardial and cerebral infarcts and vascular death [62].

Mediterranean populations have the lowest prevalence of chronic inflammatory disease and have very high life expectancy [63]. Actually, adherence to this diet is decreased [56] nevertheless many authors declare that adherence to the MedDiet has beneficial effects on diabetes mellitus (DM), obesity, and CVD [11, 64–70].

High adherence to the MedDiet reduces the overall mortality [71–73] and the risk of CVD (10%) and neoplastic diseases (4%) [71]. Adherence to MedDiet induces small favorable changes in some risk factors for CVD, but its effect on hematic lipids is generally weak [74]. Low-carbohydrate MedDiet reduces glycosylated haemoglobin (HbA1c) levels and delays the use of oral antidiabetic drugs when compared with a low-fat diet [75–77]. Recently, it has been shown that MedDiet can influence the genetics. However, there is not univocal data on health benefits [5, 6]. Importantly, investigating the rs7903146 polymorphism in the transcription factor 7-like 2 gene, Corella and coworkers [78] proved that in the homozygotes the hypercholesterolemia and hypertriglyceridemia are reduced by MedDiet.

Low adherence to MedDiet and smoking are independent predictors of 10-year CV events in women and in men, respectively [79]. The adherence to the MedDiet, nonsmoking, normal weight, and regular physical activity reduce the mortality in men and in women, but the statistical significance is reached only in women [72, 73, 80]. However, the response to the MedDiet seems to be greater in men than in premenopausal women when cardiometabolic changes are considered [81–84]. MedDiet ameliorates plasma lipid profile and diastolic blood pressure (DBP) without impacting on leptin levels and the leptin-to-adiponectin ratio in both sexes [84]. Only in men, it ameliorates the insulin homeostasis and redistribution of LDL subclasses from smaller to larger LDL, while an opposite trend is observed in women [81]. Finally, MedDiet increased telomere length, a marker of biological age, in women [85], although no consensus is found about this effect [86]. Finally, men are less adherent to MedDiet than women [87].

## 3. EVOO, VOO, OO, and MPC 

OO is produced from the fruits of *Olea europaea* L. evergreen trees, a plant cultivated worldwide, but it is typical cultivation of the Mediterranean area [88]. It mainly contains monounsaturated fats (98-99% of total weight of EVOO), such as oleic acid, followed by a low amount (1-2%) of phenols, phytosterols, tocopherols, and squalene [89]. Importantly, in EVOO only, fatty acids are stabilized by MPCs, with antioxidant activities [90].

EVOO composition and concentration in MPCs are extremely variable either qualitatively or quantitatively (200–600 mg/kg) [91]. MPCs are dependent on the tree cultivar, the climate, growing, and production procedure [92]. The phenolic cluster of EVOO can be subdivided into several subclasses. In particular, EVOO contains saponifiable compounds (triacylglycerol, partial glycerides, esters of fatty acids or free fatty acids, and phosphatides) and unsaponifiable compounds (hydrocarbons (squalene), phytosterols (*β*-sitosterol, stigmasterol, and campesterol), tocopherols, carotenoids, pigments (chlorophylls), aliphatic and triterpenic alcohols, triterpenic acids (oleanolic acid), volatile compounds, and polyphenols) [93].

In general, secoiridoids are the most representative followed by phenolic alcohols such as Tyr and HTyr, flavonoids, lignans, and phenolic acids [89, 92]. In general, HTyr, Tyr, and conjugated forms of secoiridoids like oleuropein (which are hydrolyzed to HTyr and Tyr in the stomach) are the most representative [94]. HTyr also originates by the hydrolysis of oleuropein during olive ripening or/and during the storage and elaboration of table olives [95]. It can be found in a free form, such as acetate form, or as part of oleacein, verbascoside, and oleuropein [93]. Also ligstroside, oleacein, and oleocanthal are sources of HTyr and Tyr [96].

Some of MPCs such as HTyr, Tyr, and their secoiridoid derivatives (oleuropein, oleuropein aglycone, and elenolic acid dialdehydes) are hydrophilic [97], while other MPCs are lipophilic [89]. Lignans belong to the family of phytoestrogen [98] and in general the predominant lignan is (+)-1-acetoxypinoresinol [98]. The leaves of the *Olea Europaea* L. contain higher concentrations of phenols than the olive fruit and derived oils [99–101]. The predominant MPCs in the leaves are verbascoside, apigenin-7-glucoside, luteolin-7-glucoside, HTyr, Tyr, and oleuropein [102]. Notably, a single MPC may possess distinct biological activity [103, 104]. Thus, it is impossible to extrapolate the result of the single EVOO, VOO, and OO to another. For example, Chetoui and Blanqueta cultivars (rich in linoleic acid) induce higher total triacylglycerol (TAG) incorporation into THP-1 cells than Buldiego and Picual (rich in oleic acid), promoting foam cells formation [104]. Further, extracts of Taggiasca and Seggianese, which have different amounts and composition of MPCs, have a different antioxidant activity being higher in Seggianese extract [103].

## 4. Pharmacokinetics of MPCs and Influence of Sex

The influence of sex and gender on pharmacokinetics of phenols was recently reviewed [25]. Briefly, in humans, MPCs are well adsorbed (∼40%–95%, using HTyr and Tyr as proxy) [105, 106]. It is important to recall here that, in humans, there is an endogenous synthesis of HTyr during the metabolism of dopamine with its formation being favored by ethanol [107]. In addition, HTyr is a product of oleuropein hydrolysis that can occur in the stomach. Besides, gut microbiota generates HTyr from oleuropein [108].

In the intestinal tract (both ileum and colon), more than 40% of HTyr is absorbed by bidirectional passive transport [108], which depends on numerous factors such as food matrix or vehicle. The absorption of HTyr and Tyr is higher when administered as an OO solution than as aqueous solution [108]. In the gastric and intestinal tract MPCs are hydrolyzed [109], with some exceptions. In particular, oleuropein is degraded by the colon microbiota to HTyr that is then absorbed [109]. HTyr bioavailability seems to be influenced by sex [110]. The maximum plasmatic concentration of HTyr is reached 5–30 min after administration of EVOO and VOO [108]. HTyr and its derivatives cross the blood brain barriers [111]. Finally, HTyr is incorporated in HDL, which is higher in women than in men [108].

HTyr and Tyr are extensively metabolized by phase I enzymes, such as CYP2D6 and CYP3A4, and by phase II enzymes both at intestinal and hepatic levels [108, 112]. Numerous phase I and II enzymes present numerous sex differences both in animals and in humans [33]. Thus, the metabolism of MPCs can be sex divergent at least in rats [110]. In humans, the biotransformation of HTyr and Tyr mainly occurs through glucuronidation and sulphation, and the main circulating metabolites are both HTyr sulfate and HTyr acetate [108]. HTyr is also metabolized by catechol-O-methyl transferases that are more expressed in men than in women [33] forming 3-hydroxy-4-methoxyphenyl ethanol (homovanillyl alcohol) [113]. Globally, HTyr and Tyr have lower bioavailability than their metabolites [107]. Inside the cells, the conjugated forms can be deconjugated and thus HTyr and Tyr metabolites can be reformed. Finally, the intestinal microorganisms metabolize HTyr into hydroxylated phenylacetic acid, acetic acid, and benzoic acid [114]. In plasma and urine, 98% of HTyr is recovered as glucuronide form and only 2% is free [115]. Usually, the complete elimination of HTyr and metabolites occurs approximately in 4 and 6 h in rats and humans, respectively [116]. HTyr is mainly excreted by the renal route where it is present both in conjugated and nonconjugated form [108]. Urinary HTyr levels (adjusting for ethyl glucuronide) are higher in men than in women [107]. In addition, through the biliary route they reach the small intestine where they can be retransformed and reabsorbed [116]. Despite the enterohepatic recycling, a small amount (about 5%) of total HTyr is excreted by feces [116] and the consumption of MPC-rich OO elevates the free HTyr levels in feces of men [114]. Notably, Tyr, HTyr acetate, 3,4-dihydroxyphenylacetic acid, and homovanillyl alcohol administration changes urinary excretion of catecholamines (dopamine, normetanephrine, norepinephrine, and 3-methoxytyramine) in male and female rats, with the excretion being significantly higher in male than in female rats [110].

Oleocanthal constitutes about 10% of the olive's MPCs (100–300 mg/kg EVOO) [117]. Oleocanthal, as other MPCs, is stable at acid pH and at 37°C and it is biotransformed by phase I and II enzymes, with glucuronidation being the prevalent way [117]. Oleocanthal and other secoiridoids and their metabolites are mainly eliminated by renal route and they are found in human urine 2–6 h after the intake [117].

Little and nonunivocal data are available on sex influence on bioavailability of chlorogenic acids ([118] and cited literature) and lignans. After long flaxseed lignan secoisolariciresinol diglycoside exposure, female rats have higher lignan concentrations in heart and thymus than male rats [119]. A strong association between dietary lignan intake and prevalent obesity exists only for boys [120].

Importantly, pharmacokinetic interactions with other botanicals and prescription drugs have been described. For example, bioavailability of HTyr is enhanced when co-administered with the thyme extracts [121].

Considering the role of gut microbiota in sex healthcare paradigm [122, 123] and their ability to expand metabolic activity of the host [124], it important to recall that they could be a modifier of the activity and kinetic of all compounds present in olive and leaves and other matrixes [125]. In turn, OO derivatives may influence the gut microbiome. For example, the dialdehydic form of decarboxymethyl oleuropein aglycone, oleocanthal, HTyr, and Tyr may inhibit the growth of bacteria [126], including the beneficial ones [127]. Sex-gender differences in the microbiota are recently reviewed by Kim et al. [128]. Here, it is important to recall that microbiota modifications may participate in the pathophysiology of CVD [129]. For example, some metabolites of gut microbiota such as short-chain fatty acids and trimethylamine N-oxide may participate in the modulation of blood pressure through G protein receptors [129]. Further gut microbiota may inhibit HDL-coordinated reverse cholesterol transport [129].

Globally, the effects of MPCs on microbiota appear to be compound and sex specific, and in consideration of sex differences that characterize the human microbiota, its effects on MPC fate and activity should be accurately studied.

## 5. Effect of EVOO, VOO, OO, Leaf Extracts, and MPCs on Endothelial Dysfunction: Influence of Sex

Endothelial function is a barometer of vascular health [130] and it is a predictor and a pathogenic mechanism of atherosclerosis [131], being also related to the prognosis and severity of CVD [50, 132]. Endothelial dysfunction is more precocious than atherosclerotic plaques and it is a more prominent risk factor in women than in men (Table 1). It is related to oxidative stress, inflammation, platelet activity, an alteration of glucose metabolism, and uric acid levels [133–136], and all these processes present sex differences [34, 136–140].

### 5.1. Effect of EVOO, VOO, OO, Leaf Extracts, and MPCs on Oxidative Stress: Influence of Sex

The influence of sex on oxidative stress is widely reviewed [34, 137]. However, no univocal results are obtained and this could depend on species, tissues, and cells used and on donor age. For example, Brunelli et al. [141] report no differences in the plasma antioxidant barrier, although women present a higher oxidative status than men. Moreover, they suggest that premenopausal and postmenopausal women are similar [141]. By contrast, Vassalle and coworkers [47] report that menopause is a condition that elevates oxidative stress. Further, young men have lower levels of malondialdehyde (MDA) in comparison to fertile women and older men [142]. After correction for body weight (BW), both pre- and postmenopausal women have higher amounts of carbonylated proteins *vs* men of similar age [142]. Others show that lipid and protein oxidation are increased in peri- and postmenopausal women, whereas superoxide dismutase (SOD) and catalase (CAT) activities are decreased and increased in postmenopause and in perimenopausal women, respectively [143]. Glutathione (GSH) and glutathione peroxidase (GPx) are lower in women aged 32–39 years than in women aged 20–25 years. Meanwhile, 20–25-year old men have higher GSH and lower glutathione disulfide (GSSG) than women of the same age. The SOD and CAT activities are higher in women aged 32–39 years than in men and women of younger age [144]. Moreover, women with CAD seem to have higher oxidative stress than men [145]. Another study shows that African American women with symptomatic peripheral artery disease produce more ROS than men, while Caucasian men and women do not diverge indicating that ethnicities could play a role in sex and gender differences [146–150]. Others report the opposite trend and others do not find any significant sex difference [151–153].

The antioxidant activity of EVOO, VOO, and MPCs is extensively reviewed [154, 155] (Table 2). It is based on their scavenger, chain breaking, and chelating activities [116]. Moreover, they favor the resistance over oxidation [266]. High dose of oleuropein and HTyr may exert prooxidant activity [267, 268], and this paradoxically could be one of the mechanisms of their antioxidant activity because it can activate the translocation of nuclear factor E2-related factor 2 (Nrf2) to the nucleus [269] in a sex-specific manner [270, 271] that leads to modifications of proteins expression and activity such as *γ*-glutamylcysteine ligase, which is expressed less in female rat livers than in male ones [272]. After trauma and hemorrhage, HTyr elevates liver Nrf2 modulating heme oxygenase-1 (OH-1) especially in rat females (proestrous phase) compared to males [273]. Through Nrf2, MPCs can also activate phase II detoxifying enzymes and mitochondrial biogenesis, two critical pathways in reducing the negative effect of oxidative stress [271]. Oleuropein and HTyr seem to be scavengers of HOCl [274], which starts LDL lipid peroxidation and oxidizes the apolipoprotein (Apo) B-100 [275]. However this is not a univocal result [213]. Finally, in animals and in humans, HTyr may interact with several microRNAs [218, 276] that regulate numerous cellular function including DICER function that is relevant to the redox state [277, 278].

### 5.2. Effect of EVOO, VOO, OO, Leaf Extracts, and MPCs on Inflammatory Response: Influence of Sex

The effect of sex on inflammatory response has been recently reviewed [138, 139, 279]. Women and men have a different immune system [281] and arachidonic acid (AA) cascade [281]. This last generates numerous compounds with proinflammatory and anti-inflammatory activities. Interestingly, females seem to be protected against endothelial dysfunction induced by systemic inflammation [282]. In particular, COX2 and COX1 female knockout mice have less inflammatory edema and joint destruction than male mice [283]. Consistently, expression of COX2 is more elevated in male than in female cells [284]. More PGE_2_ is produced by human male neutrophils *vs* female ones [284]. In male coronary rat arteries, PGF_2_*α* exerts a major contraction in male arteries than in female ones for the presence of more PG receptors [285]. Also the lipoxygenase (LOX) system presents some sexual dimorphism. 5-LOX and its 5-lipoxygenase-activating protein (FLAP) are downregulated by androgens [286]. Thus, the bigger production of leukotrienes in monocytes and neutrophils of women is not surprising [286]. In human neutrophils and monocytes, the synthesis of lipoxin A_4_ (LXA_4_), a proresolving molecule [287], is reduced by estradiol [281]. Further, a positive and a negative correlation exist between age and aspirin triggered 15-epi-LXA_4_ in women and men, respectively [288]. Resolvins, protectins, and maresins activities may be influenced by sex [289]. For example, D-resolvin is higher in women exudate whereas chemoattractant leukotriene B_4_ is higher in men [282]. The precursors of oxylipins are higher in the female urine than in male one [290].

Also the nuclear factor-kappa b (NF-*k*B) pathway, which is crucial for inflammatory response [291], is sex-dependent with its activation being mediated by the adaptor molecule MyD88, which interacts with cytoplasmic estrogen receptor-*α* [292]. The NF-*k*B activation is higher in female human umbilical cord vein endothelial cells (HUVEC) than in male ones, under hyperoxic conditions [293]. Also the tumor necrosis factor-*α* (TNF-*α*) pathway exhibits sex differences. For example, the human female adult cardiac progenitor cells appear to be more responsive to TNF-*α* when migration and cell cycle progression are considered [294]. Young men have lower levels of TNF-*α* when compared to fertile women [142]. Also the interleukin systems present some sex differences, with IL-6 being significantly higher in postmenopausal women than in premenopausal women [142], and in young women with CAD either in basal condition or after stress than men [295]. The anti-inflammatory effects of OO and its derivatives are summarized in Tables 2 and 3. In general, female animals and women are less studied and OO with a high content of MPCs is more active in the control inflammation, redox status, and lipid metabolism than OO with low content of MPCs. For example, EVOO with high MPCs reduces peripheral blood mononuclear cells (PBMC) activation of the CD40/CD40 ligand (CD40L) and LDLox and modifies numerous genes [313]. Some MPCs like HTyr exert anti-inflammatory activity with multiple mechanisms attenuating iNOS, COX2, and IL-1*β* expression and TNF-*α* and inhibiting the activation of granulocytes and monocytes [116]. Also oleocanthal and Tyr inhibit COX [246, 360].

### 5.3. Effects of EVOO, VOO, OO, Leaf Extracts, and MPCs on Platelets Function: Influence of Sex

Human platelets are sexually divergent; women have more platelets, longer bleeding time, and more activatable glycoprotein IIb/IIIa than men whereas platelet spreading and adherence are higher in men than in women [135]. The already described sex differences in AA pathways may induces sex differences in platelet aggregation. Adenosine diphosphate (ADP) and collagen-induced aggregation are higher in women, and women and men respond differently to antiaggregating agents [135, 361]. Both preclinical and clinical studies (Tables 2 and 3) show that EVOO and some of its MPCs (HTyr, oleuropein aglycone, luteolin, and oleocanthal) reduce platelet aggregation [13, 180], interfering either with AA pathways [362] or with other mechanisms such as calcium mobilization and attenuating iNOS activity [247, 363]. In hypercholesterolemic patients, MPCs decrease platelet aggregation inhibiting procoagulant factors, such as plasminogen activator inhibitor-1 and factor VII [364]. Small crossover trial proves that oleocanthal is the most active in inhibiting collagen-induced aggregation at least in men [180], probably because it is a nonselective inhibitor of COX. HTyr antiaggregant activity seems to be agonist specific [209]. However, in vivo, it remains difficult to discriminate EVOO associated effects of specific MPCs and phenols. Tables 2 and 3 show that, globally, the majority of the studies are performed on males and even when females are recruited no sex analysis is performed.

### 5.4. Effect of EVOO, VOO, OO, Leaf Extracts, and MPCs on Glucose Metabolism: Influence of Sex

Their effects are summarized in Tables 2 and 3. Briefly, the antidiabetic actions may reside in the inhibition of *α*-amylase and *α*-glucosidase [166, 167, 214, 365], which might lead to less effective absorption of glucose [366]. Some authors suggest that HTyr is a better inhibitor of *α*-amylase than of *α*-glucosidase [214]. Also oleuropein inhibits these enzymes [214]. Beyond the inhibition of these enzymes, other mechanisms have been proposed for the antidiabetic activity including antioxidant and anti-inflammatory action (see above) and activation of AMP-activated protein kinase and of incretin release [197, 205–207, 341]. In particular, the antidiabetic activity of HTyr and oleuropein is recently reviewed [367, 368]. Again it emerges that the antidiabetic activity has been mainly studied in males; nevertheless, it clearly shows that DM presents numerous sex differences [39], including the relative risk for CVD associated with hyperglycaemia that is higher in women than in men (Table 1).

### 5.5. Effects of EVOO, VOO, OO, Leaf Extracts, and MPCs on Uric Acid: Influence of Sex

It is related to CV events both in women and in men [140, 369, 370], but it is a higher risk in women [371]. However, these are not univocal data because others sustain that this association is present only in women [372–374], who have lower plasma levels that men [375]. Leaf extracts of olive tree and HTyr inhibit xanthine oxidase reducing uric acid synthesis [376]. In male rats, HTyr also regulates transcription of some renal transporters that favor uric acid excretion [377].

## 6. Clinical Studies

Results of clinical studies are summarized in Table 3. The beneficial aspects of regular use of OO on CVD has been suggested by numerous authors [2, 154, 306, 310, 378–380], through the biological activities discussed above and summarized in Table 2. However, clinical studies have common limitations: (a) despite the numerosity of studies, the size of samples is very small and they do not take into account the high interindividual variability; (b) they are relatively limited or of questionable quality; (c) with some exceptions they are very short in duration; (d) they are mainly performed on Mediterranean populations; (e) they have heterogeneous designs, with variation in control diets and in the type of oil used. Therefore, to overcome these limitations we focus on meta-analyses.

Schwingshackl and Hoffmann [381] reported that the use of OO is associated with a 20–40% lower risk of stroke and CHD. Another meta-analysis of case-control, prospective cohort studies and randomized controlled trials proves a negative relationship between OO consumption and stroke (and stroke and CHD combined), but the association is not significant for CHD [348]. A successive meta-analysis proves that high EVOO MPCs ameliorate surrogate end points such as lipid peroxidation, oxLDL, Chol, and HDL [382]. In addition, the subgroup analysis indicates an improvement in inflammatory biomarkers and in BP [382]. After pooling oil interventions, PCR and IL-6 are lowered compared to baseline [380]. Others show that the regular dietary intake of OO reduces CRP, IL-6, and TNF-*α* [383]. The comparison of the effect of different types of OO (refined, mixed, low, and high MPC EVOO) shows no significant effects on Chol, HDL, TG, or DBP [3]. However, in secondary analyses, EVOO may reduce oxLDL *vs* refined OO in a dose-dependent manner. Finally, one meta-analysis that includes 1089 participants shows that OO increases HDL reducing LDL and TG, while ApoA1 and ApoB are not significantly changed [384].

Another crucial risk factor for CVD is hypertension [385], a condition that presents numerous sex differences [386]. After 4 years of follow-up, results of interventional and randomized PREDIMED study show no significant variations in systolic blood pressure (SBP), whereas DBP is decreased in EVOO and EVOO + nuts MedDiet [387]. The 1-year trial that examines 235 subjects (56.5% women) proves that MedDiet supplemented with either EVOO or mixed nuts reduces SBP and DBP [388]. A meta-analysis, which includes primary and secondary prevention trials proves that high MPC OO slightly reduces SBP and oxLDL compared to low MPC OO, leaving Chol, TG, MDA, and DBP unchanged [389]. A very small decrease in blood pressure is observed in MedDiet + EVOO or nut *vs* a low-fat control group [390]. Finally, the meta-analysis of RTC of PREDIMED shows that the MedDiet lowers SBP by 3.02 mm Hg and DBP by 1.99 mm Hg [391]. Importantly, a systemic review that includes primary prevention proves the importance of pharmaceutical form because only liquid oil but not capsule with oil significantly reduces DBP [392].

OO impacts on glucose metabolism, two meta-analyses, which include cohort and interventional studies in prevention and care of DM2 [380, 393], prove that there is a 16% risk reduction in people that consume more OO with high amount of MPCs *vs* those who consume OO with small amounts of MPCs. In patients with DM2, OO supplementation reduces HbA1c, fasting plasma glucose and inflammatory biomarkers, compared to controls [380]. In addition, MedDiet and MedDiet + EVOO + nuts reduce metabolic syndrome and insulin resistance in the postpartum [394, 395].

Indeed, a systemic review and meta-analysis, which includes RC trials that examine lipid profile, inflammation, and oxidative stress biomarkers in individuals that consume low MPC OO and high MPC OO, observed the improvement in MDA, oxLDL, Chol, and HDL. The subgroup analyses and individual studies measure additional improvements in inflammatory markers and blood pressure. Nevertheless, the authors conclude that there is a need for longer-term studies in non-Mediterranean populations because most studies were rated as having low-to-moderate risk of bias [382]. A recent meta-analysis, including RC trials for more than 3 weeks and examining at least two of the following OO: refined OO, mixed OO, low MPC EVOO, and high MPC EVOO, suggests that it is not possible to reach any clear conclusion for the beneficial effects [3]. Moreover, in line with what was observed with prescription drugs [39], a gender gap exists because the majority of clinical studies are performed mainly on males, and if they include females, results are not stratified for sex. This leads to low scientific value of the results in consideration of the numerous sex differences observed in CVD, DM, and hypertension (Table 1).

## 7. Conclusions

To have a clear conclusion, it is important to harmonize study design. For example, it will be important to declare whether the goal is the use of OO as a supplement or as a part of dietary pattern. If it is given as a supplement, it is important to consider the pharmaceutical form (liquid, capsule, and excipients) because this could modify both the pharmacokinetics and pharmacodynamics. Furthermore, considering the prevention and therapy of noncommunicable diseases such as CVD and DM, there is a need for long-term studies that consider also a sufficient number of extra-Mediterranean people and low-risk populations (most of the trials are conducted on high-risk populations and this could result in underestimation of possible benefits on low-risk populations [396]).

Considering the great sex differences observed in CVD (Table 1) and in DM [32, 39, 397] and the possible sex-divergent effects of MPCs [25, 26, 398, 399], it is necessary to enroll males and females in studies, to overcome the sex and gender gap that pervades all the research in the field of the OO, VOO, EVOO, leaf extracts, and MPCs. In the era of personalized medicine, it is mandatory to consider the sex and gender aspects to answer a multiplicity of questions regarding the effects of diet and specific diet components on health and to relieve consumer uncertainty and promote health, comprehensive cross-demographic studies using the latest technologies, which include foodomics, integrated omics approaches, personomics, and appropriate study design.

## Figures and Tables

**Table 1:** Examples of sex and gender differences in CVD and risk factors.

Diseases or risk factors	Sex differences	References
Myocardial infarction	Women are 10 years older than males and have higher mortality in younger ages and have more atypical symptoms. Women have less anatomical obstructive CAD than men; it is estimated a 20% or greater excess of normal or nonobstructive arteries in women vs men	[40–42]
Heart failure	Lower incidence in women but the prevalence is similar in both sexes, with diastolic heart failure being more common in women. Lower mortality rate in women than in men	[40, 41]
Hypertension	Lower incidence in premenopausal women	[40]
Cardiac hypertrophy	Premenopausal women are better protected than men; men have more cardiac hypertrophy	[40, 43]
Ischemia-reperfusion injury	Studies evidenced that females have lower ischemia-reperfusion injury	[40]
Diabetes	Higher increased risk of CVD in women vs men	[40]
Endothelial dysfunction	More frequent in women vs men	[44, 45]
HDL	Higher levels in women vs men; the difference declines with age	[46]
TG	Higher increased risk of CVD in women vs men. In women, they increase after menopause	[47]
Chol	Levels rise in menopausal transition period	[47]
LDL	Levels rise in menopausal transition period	[46]
Lp (a)	Levels rise in menopausal transition period	[46]
Smoking	Less women smoke vs men, but smoking has more negative effects on women	[48]
Social economicus status	In women, it is inversely associated with increased risk of CAD, stroke, and CVD. In particular, for CHD, it is associated with lower education	[49]
Psychological factors	Women had higher contributions from psychosocial risk factors (45.2% vs 28.8% in men)	[50, 51]
*Unique for women*		
Gestational diabetes, pre-eclampsia, syndrome of polycystic ovary	Higher increased risk of CVD in women	[48, 52]
Oral contraceptives	A large cohort study (1.6 million of women, 15 to 49 years old) shows that ethinylestradiol (20 *μ*g or 30 to 40 *μ*g) is associated with an increased risk of MI. The risk is not significantly varied by progestin	[53]
	OC should not be prescribed for women over the age of 35 years and smokers (American College of Obstetricians and Gynecologists) and should be prescribed with caution in case of CV risk factors such as hypertension, diabetes, and dyslipidemia	[54]
Hormone replacement therapy	A large cohort study shows that ethinylestradiol is associated with an increased risk of MI that is not significantly changed with progestins	[55]

**Table 2:** Some CV effects of EVOO, VOO, OO, leaf extracts, and MPCs.

EVOO, VOO, OO, leaf extracts, and MPCs	Activity	References
Acetoxypinoresinol	Using DPHH test, it exerts antioxidant effects	[156]
Caffeic acid	It inhibits 5-LOX and exerts an antioxidant effects in *male* rat peritoneal leukocyte triggered by calcium ionophore and PMA	[157]
	It decreases IL-1*β* in human blood cultures (sex not reported) stimulated with LPS	[158]
EVOO	*In healthy men*, EVOO reduces urinary excretion of urinary 8‐oxo‐deoxyguanosine by 13%	[159]
	In *30 hamster males*, it reduces atherosclerosis	[160]
	In ApoE deficient mice *(14 females and 22 males),* the antiatherogenic effect of EVOO is reduced by dietary cholesterol	[161]
	In ApoE deficient mice (*54 females*), EVOO from different cultivars reduces atherosclerotic lesions, plaque size, and macrophage recruitment if compared to diets containing palm oil. EVOO also induces a cholesterol-poor, ApoA-IV-enriched lipoparticles with enhanced arylesterase and antioxidant activities	[162]
	In *male* STZ-diabetic rats, it raises BW and HDL and decreases glycaemia, TG, Chol, being ineffective in healthy rats	[163]
	In STZ-diabetic rats (*sex not reported*), it elevates HDL and reduces Chol, TG, and LDL	[164]
	In human platelets obtained from *3 male and 2 female* healthy subjects, it reduces NOX2 activation and H_2_O_2_ production	[165]
	In vitro, it inhibits ACE, *α*‐glucosidase, and *α*‐amylase being more active vs *α*‐glucosidase; the richest MPC EVOO is also the most active	[166]
	Seggianese EVOO extract (rich in secoiridoids) is more active in preventing human LDL oxidation than Taggiasca EVOO extract (rich in lignans) (*sex not reported*)	[103]
	In vitro, Spanish EVOO inhibits *α*-glucosidase, *α*-amylase, and 5-LOX	[167]
	LDL and HDL obtained from treated healthy *14 women and 10 men* are less oxidizable and are more resistant to lipid peroxidation. Both EVOO and EVOO extract enhance the Chol efflux	[168]
	In *male hypertensive rats*, EVOO + olive + leaf rich in HTyr, 3,4 dihydroxyphenylglycol, and oleuropein decreases BP, angiotensin II, and endothelin-1 *vs* low MPC oil. There are no significant differences in plasma Na^+^, urea, HDL, and LDL	[169]
	In an acellular model, HTyr rich extracts have a higher antioxidant and antimutagenic activity than Tyr-rich extract. In HELA cells, the Tyr-rich extract is more effective in increasing GSH whereas ROS levels are not changed by tested EVOO extracts. All extracts upregulate Keap1/Nrf2 pathway	[170]
	*In male mice*, high-fat EVOO diet improves glycaemia, insulinemia, glucose tolerance, insulin sensitivity, and insulin secretion. It reduces *β*-cell apoptosis and normalizes islet glucose metabolism vs high fat lard diet	[171]
	EVOO extract inhibits p50 and p65 NF-kB translocation in both stimulated and unstimulated PMA-challenged human monocytes and monocyte-derived macrophages (*sex not reported*)	[172]
	In ECV304 cells (*sex not reported),* EVOO extract partially prevents the increase of NO/ET-1 levels induced by high glucose/FFA	[173]
	In *male rats*, a bolus of EVOO changes the phospholipids of HDL	[174]
	Serum obtained from 6 healthy *males* and 6 *females* treated with EVOO extract rich in oleuropein and ligstroside reduces the VEGF-stimulated increase in NOX, Nox4, and MMP-9 activities, migration, and invasiveness. It also regulates VEGF-induced morphological differentiation capacity of HUVEC *(sex not reported)* into capillary-like structures. In human microvascular endothelial cell line, it reduces the VEGF-induced angiogenesis	[175]
	In *male* rats, subacute administration of both EVOO rich in MPC and native EVOO with low MPCs reduces ADP platelet aggregation, but acutely only MPC-rich extract reduces ADP induced aggregation	[176]
	In vitro unfiltered EVOO extract with peptide of low molecular weight inhibits ACE angiotensin converting enzymes in vitro, and in hypertensive *male* rats, it reduces SBP and DBP	[177]
	In ApoE deficient mice (*sex not reported*), extracts (EVOO vs EVOO + polyphenols green tea) enhance macrophage Chol efflux but only EVOO + polyphenols green tea reduces lipid peroxidation	[178]
	In vitro, Galician EVOO with high level of oleuropein and ligstroside derivatives inhibits the *α*-amylase and *α*-glucosidase, being more effective in inhibiting *α*-glucosidase than acarbose	[167]
EVOO vs sunflower oil, sunflower oil + oleic acid, MPC-deprived EVOO, sunflower oil enriched with the MPC of EVOO, and sunflower oil + oleic acid + MPC of EVOO	In all *male* rats fed with a high-Chol diet, GSH and IL-6 do not vary. EVOO, sunflower oil + MPC of EVOO, and sunflower oil + oleic acid + MPC of EVOO decrease the elevation in MDA and TNF-*α* levels induced by high-Chol diet	[179]
OC-rich EVOO with 1 : 2 oleacein/oleocanthal, 2 : 1 (D2_i_2) rich in Tyr; EVOO 1 : 2 oleacein/oleocanthal (D2_i_0.5) rich in Tyr	In healthy men (20 and 50 years), 40 ml of enriched EVOO for one week reduces collagen-stimulated platelet aggregation	[180]
OO	*In ApoE deficient mice (77 males 63 females),* all treatments reduce TG being ineffective versus Chol and vs the number of lesions; however, their dimensions are reduced in females by palm and olive II oils	[181]
	In *40 male* new Zeeland rabbits, dietary supplementation with 15% OO reduces the thrombogenic factors and elevates antithrombotic factors	[182]
	In *male rats*, OO reduces and prevents the growth of urinary stones	[183]
VOO	In *24 male* new Zeeland rabbits, it reduces atherosclerosis	[184]
	In *40 male* new Zeeland rabbits, it reduces atherosclerosis	[182]
	In human PBMC *(sex not reported)* and HL60 cells *(sex not reported*), it inhibits H_2_O_2_ and PMA induced DNA damage, being HTyr and Tyr, respectively (extract without verbascoside)	[185]
Extract of olive cake vs extract of thyme and vs extract of olive cake + thyme extract	In *male* rats, single oral administration of the three extracts regulates plasma antioxidant status (DPPH and FRAP) in a time and extract dependent way. In red cells, extracts decrease SOD but increase GPx and CAT	[186]
HTyr	In vitro experiments, HTyr and many other phenolic compounds added to standard cell culture media (such as DMEM, MEM, or RPMI) produce H_2_O_2_ in the one- to three-digit micromolar range	[187, 188]
	In alloxan-diabetic *male rats*, it lowers glycaemia, TG, Chol, alkaline phosphatases, AST and ALT, aspartate and lactate transaminases, lipid peroxidation, total and direct bilirubin, creatinine, urea and increases HDL and hepatic and renal SOD, CAT, and GPx	[189]
	In alloxan*-diabetic male rats*, it decreases glycaemia, Chol, and oxidative stress	[190]
	In STZ-diabetic *male rats*, it reduces plasma lipid peroxidation, nerve conduction velocity, and thermal nociception and attenuates the decline of sciatic nerve Na^+^K^+^ ATPase activity	[191]
	In STZ-diabetic *male rats,* it lowers oxidative, nitrosative, and inflammatory biomarkers and platelet aggregation	[192]
	In STZ-diabetic *male rats*, it reduces retinopathy, lipid peroxidation, nitrosative stress, TBX2, 6-keto-PGF1*α*, and IL-*β*1	[193]
	In STZ-diabetic *male rats*, it lowers retinal ganglion cell number, retinal thickness, and cell size	[193]
	In STZ-diabetic *male rats*, it reduces brain lipid peroxidation and inflammation, nitrosative stress, cell death, IL-1*β*, PGE_2_	[194]
	In STZ-induced diabetic and triton WR-1339 induced hyperlipidemic *male mice*, it reduces plasma glucose, TG, Chol, lipid peroxidation, TNF*-α*, CRP and elevates, glucose tolerance, antioxidants, and atherosclerotic index	[195]
	It prevents metabolic syndrome and inhibits the hepatic and muscular SREBP-1c/FAS pathway reducing oxidative stress and mitochondrial abnormalities and improving lipid and glucose metabolism in *db/db C57BL/6J male mice*	[196]
	In the brain of diabetic *db/db C57BL/6J male mice*, it activates AMPK, SIRT1, and PPAR*γ* coactivator-1*α* and reduces oxidative stress	[197]
	In LPS-stimulated human monocytic cells (*sex not reported*), it suppresses NO release and attenuates the transcription and expression of TNF-*α*, iNOS, and COX2 in a dose-dependent way	[198]
	In HUVEC (*sex not reported),* HTyr and its metabolites suppress TNF-*α*-induced phosphorylation of NF-*κ*B, ROS production, depletion of GSH, adhesion molecules and downregulate genes encoding antioxidant enzymes. They also reduce the adhesion of human monocytes (cell line) to HUVEC. Finally, they reduce carrageenan induced paw edema and TPA-induced ear edema *in male* mice	[199]
	The HTyr pretreatment of HUVEC (*sex not reported*) suppresses inflammatory angiogenesis induced by PMA and ameliorates mitochondrial function	[200]
	In *male* mice, it ameliorates the impact on body adiposity induced by the obesogenic diet	[201]
	In *male rats fed* fed with high-fat diet, it reduces AST, ALT, Chol, liver inflammation, and nitrosative/oxidative stress. It improves glucose tolerance, insulin sensitivity, and intestinal barrier integrity and functions and increases hepatic PPAR*α* and its downstream-regulated genes	[202]
	In *male* mice fed with diet-induced obesity, it improves glucose homeostasis, insulin signaling markers, chronic inflammation, hepatic steatosis, and endoplasmic reticulum stress	[203]
	In *male* rats fed with a diet-induced metabolic syndrome, it reduces adiposity and ameliorates impaired glucose, insulin tolerance, and endothelial dysfunction. It also decreases SBP, left ventricular fibrosis, and resultant diastolic stiffness and markers of liver damage. Notably, the diet used for induction of metabolic syndrome alters HTyr metabolism	[204]
	In endothelial cells obtained from porcine pulmonary arteries (*sex not reported*), it increases AMPK, CAT activities, forkhead transcription factor, and cytoprotection against TNF-*α*-induced damage through the suppression of caspase-3 and NF-*k*B activation. It also promotes wound healing via Nrf2 synthesis and stabilization	[205, 206]
	In rat aorta VSMC (*sex not reported*), it exerts a proapoptotic effect through NO production and protein phosphatase 2A activation with subsequent inactivation of AKT	[207]
	In *male* rat peritoneal leukocytes triggered by calcium ionophore, it inhibits 5-LOX and exerts antioxidant effects in leukocytes triggered by PMA	[157]
	In a *female mice* model for accelerated aging, it induces the expression of SIRT1	[208]
	In vitro, it inhibits human platelet (*sex not reported*) aggregation induced by ADP and collagen being more active than other MPCs and TBX2 production induced by collagen and thrombin	[209]
	In pooled human liver microsomes (*sex not reported*), it inhibits androstenedione 6*β*-hydroxylase and reductive 17*β*-HSD activity, whereas it is inactive *vs* oxidative 17*β*-HSD	[210]
	In white adipose of *male mice* fed with high-fat diet, it reduces the increase in oxidative stress, lipid, and protein oxidation and increases the antioxidant defenses	[211]
	In adult *male* rats, it reduces myocardial infarction area, necrosis and apoptosis, the release of LDL and CPK, probably through upregulation of PI3K/AKT pathway	[212]
	It is a scavenger of hydroxyl radicals, with peroxynitrite and O_2_^−^ being inactive *vs* HOCl and H_2_O_2_. It protects LDL against oxidation but is not effective vs the oxidation of LDL isolated from humans after HTyr intake (*sex not reported*)	[213]
	It inhibits *α*-glucosidase and *α*- amylase, being more effective vs *α*-glucosidase	[214]
	In human aortic endothelial cells (*sex not reported*) stimulated with TNF‐*α*, it significantly reduces the secretion of P‐selectin, ICAM‐1, VCAM‐1, and MCP‐1	[215]
	In human HUVEC *(sex not reported),* it reduces the stimulated tube-like differentiation and the stimulated locomotion, MMP-9 secretion induced by PMA, PMA-stimulated COX2 activity and expression. Pretreatment with HTyr before PMA decreases intracellular ROS and nuclear translocation of the p65 NF-*κ*B subunit and NF-*κ*B transactivation	[216]
	In *male rats*, HTyr, 3,4-DHPEA-EA and 3,4-DHPEA-EDA reduce the increase in intracytoplasmic Ca^2+^ induced by vasopressin. Further, higher concentration of HTyr exerts an endothelium-independent effect. 3,4-DHPEA-EA and 3,4-DHPEA-EDA exert an endothelium-dependent vasodilation in aorta increasing the production of NO	[217]
	It regulates expression of numerous miRNA in the mice gut (*sex not reported)* being less effective in other tissues. HTyr administration increases TG	[218]
	*In male mice*, it lowers Chol	[219]
	In human monocytes (*sex not reported*) stimulated with PMA, it reduces the expression of mRNA and protein of COX2 decreasing PGE_2_ and O_2_-production and increases TNF-*α* production. In human neutrophils (*sex not reported*) stimulated with PMA, or chemotactic peptide FMLP or opsonized zymosan particles, it does not influence the production of O_2_− and NOX activity whereas it inhibits the production of H_2_O_2_	[220]
	In human PBMC (*sex not reported*) and in human monocytic cell line U937 stimulated with PMA, it reduces the secretion of MMP-9, PGE_2_ production, COX2 protein expression, and COX2 mRNA without modifying COX1. It inhibits both PGE_2_ and MMP-9 release from human monocyte-derived macrophages. It suppresses NF-*κ*B activation in human monocytoid cells and reduces PKC*α* and PKC*β*1 activation. Notably, it does not affect MMP-9 and COX2 in basal conditions	[221]
	In LPS-stimulated human monocytic THP-1 cells (*sex not reported*), it reduces LPS-stimulated NO and ROS formation in a concentration-dependent way, increases GSH levels, and suppresses the of NF-kB activation	[222]
	In young *male* C57BL/6 mice treated with MPC does not modify BW, food intake, and TG but it lowers plasma Chol, leptin. In murine 3T3-L1 preadipocytes, it positively modulates the glutathione-driven antioxidant enzymatic machinery reducing GSSG/GSH ratio, through the modulation of genes related to oxidative stress	[223]
	In *male rats* with diet-induced metabolic syndrome, it decreases glucose tolerance, lipids, ALT, AST activity, insulin, weight gain, fat mass, liver steatosis, and ventricular fibrosis	[204]
	It prevents COX2, TNF-*α*, DNA damage, and oxidative stress in Balb/c mice treated with LPS (*sex not reported)*	[224]
	It increases the TNF-*α* mRNA level in LPS-activated human monocytes (*sex not reported)*	[225]
HTyr, oleuropein, EVOO extract, homovanillyl alcohol	In HUVEC (*sex not reported)*, EVOO extracts decrease cell surface expression and mRNA of ICAM-1 and VCAM-1. Olea and HTyr are the main actors for these effects. Homovanillyl alcohol inhibits cell surface expression of adhesion molecules, but the effects on mRNA are small	[226]
HTyr HTyr- acetate (HTyr-Ac) HTyr ethyl hydroxytyrosol ether (HTyr-Et)	In *male* rats fed with high-fat diet, the compounds improve glucose, insulin, leptin levels, lipid peroxidation, and antioxidant capacity status, with HTyr-Ac being the most active. They also reduce the release of inflammatory biomarkers. HTyr-Ac and HTyr-Et improve adipose tissue distribution and adipokine production, decreasing MCP-1 and IL-1*β* levels	[227]
HTyr and homovanillic alcohol	In PBMC obtained by *healthy men and women*, they inhibit the increase of IL‐1*β*, MIF, and RANTES induced by oxysterols	[228]
HTyr-acetate (HTyr-Ac)	In TNF-*α*- stimulated HUVEC (*sex not reported*), it reduces the inflammatory response partly through the TNFRSF1A/SIRT6/PKM2-mediated signaling pathway	[229]
HTyr and oleuropein	Both compounds inhibit oxidative burst in human granulocytes and monocytes obtained from healthy individuals (*sex not reported*) stimulated with PMA. HTyr attenuates the generations of NO and PGE_2_. In LPS triggered RAW264.7, it reduces NRf2 nuclear translocation and miR-146a expression	[230]
HTyr and HTyr-NO	In vascular ring obtained from *male rats*, it releases NO while HTyr is ineffective. HTyr NO decreases Chol, TG, lipid peroxidation and increases SOD and NO in the serum of STZ-diabetic *male mice*. Both HTyr-NO and HTyr upregulate SIRT1 expression in the thoracic aorta of *male* diabetic mice. In HUVEC triggered by hyperglycaemia (*sex not reported*), HTyr-NO increases cell viability and reduces oxidative stress trough SIRT1	[231]
HTyr, dialdehydic form of elenolic acid linked to HTyr, oleuropein aglycon, oleuropein, Tyr, the dialdehydic form of elenolic acid linked to Tyr, caffeic acid, and verbascoside	In human PBMC and HL60 cells *(sex not reported)*, they inhibit H_2_O_2_‐induced DNA damage	[185]
HTyr + nicotinate	It inhibits *α*-glucosidase, and in healthy *male* mice fed with high-fat diet, it has hypoglycemic, antioxidant, and hypolipidemic activities	[232]
HTyr + eicosapentaenoic acid (EPA)	In *male* mice fed a with high-fat diet, it reduces the steatosis and elevates the hepatic levels of eicosapentaenoic acid (EPA), docosahexaenoic acid (DHA), resolvins and attenuates proinflammatory markers	[233]
Leaf extract	In INS-1 cells (*sex not reported*), leaf ethanolic extract and oleuropein improve the damage induced by H_2_O_2_. The leaf extract is more potent than oleuropein in preventing the cytotoxic effects and only leaf extract preserves GPx	[234]
	In STZ-diabetic *male* rats, the extract ameliorates diabetic alterations	[235]
	In STZ-diabetic *male* rats, it decreases glycaemia and HbA1c and increases insulin. It also inhibits *α*-amylase and *α*-glucosidase	[236]
	In acellular model, it inhibits DPPH radical generation. In STZ-diabetic *male* rats, the extract increases CAT activity, GSH and lowers lipid peroxidation, Chol, TG, histological pancreas, and hepatic damage	[237]
	In *male* alloxan-diabetic rats, it shows a hypoglycemic effect and reduces the damage of islets of langerhans	[238]
	In cultured neonatal rat cardiomyocytes *of both sexes*, it decreases maximum I (Ca,L) in a reversible manner	[239]
	*Male rats* fed with a high-fat diet develop signs of metabolic syndrome. Comparing rats with high-fat diet *vs* those with high-fat + leaf extract enriched with MPCs, it emerges that leaf extract improves the signs of metabolic syndrome and decreases MDA and uric acid while it is not effective on BP	[240]
	In human coronary artery endothelial cells (*sex not reported*) stimulated with serum amyloid A, it reduces the release of IL-6, IL-8, mRNA expression of E-selectin, the phosphorylation of p65 of NF-*κ*B, DNA damage and stabilizes microRNA-146a and let-7e	[241]
	In *male rats*, leaf extract containing 20% of HTyr decreases the paw edema induced by carrageenan and IL‐1*β* and TNF‐*α* release. It does not affect the anti-inflammatory cytokine IL‐10	[242]
	In diet-induced hypercholesterolemic *male rats,* olive leaf extracts enriched with oleuropein enzymatic and acid hydrolysates rich in oleuropein aglycone and HTyr decrease Chol, TG, and LDL and elevate HDL and serum antioxidant potential. In livers, hearts, kidneys, and aorta lipid peroxidation decreases while liver CAT and SOD increase	[243]
Luteolin	It is antioxidant in chemical test and prolongs the lag phase of LDL oxidation. It protects the cells against H_2_O_2_ induced damage but it is ineffective *vs* platelet aggregation (*sex not reported*)	[209]
Oleacein	In vitro, it inhibits angiotensin converting enzyme	[244]
	It stabilizes atherosclerotic plaque in samples obtained from 20 hypertensive individuals of *both sexes*	[245]
Oleocanthal	It is a nonselective inhibitor of COX1 and 2 and attenuates iNOS and human recombinant 5-LOX, being ineffective *vs* 15-LOX. Regarding 5-LOX, it is less active than oleuropein and oleacein. In addition, it inhibits TNF-*α*, IL-1*β*, IL-6, and GM-CSF	[117, 246, 247]
	In rat and mouse trigeminal ganglia (*females and males used in equal ratio*), it acts as agonist of TRPA1	[248, 249]
	In *male* adult rats, it decreases the traumatic injury reducing the inflammatory response by reducing the eNOS and iNOS	[250]
	In murine chondrogenic ATDC-5 cells and in mouse macrophage J774A.1, it inhibits the LPS-mediated upregulation of NOS2 and LPS induced release of cytokines (*sex not reported*)	[251]
	In human monocytes (*sex not reported*), it reduces the release of O_2_^−^, PGE_2_ and the expression of COX2 and inhibits NAPH-oxidase	[220]
Oleuropein	In vitro, it inhibits *α*-glucosidase and *α*- amylase	[214]
	In C2C12 cells (*sex not reported*), it protects against H_2_O_2_ induced damage; further it increases glucose consumption and the phosphorylation of AMPK/ACC and MAPK, but not PI3 kinase/Akt. It improves the insulin sensitivity via insulin-dependent (PI3 kinase/Akt) and insulin independent (AMPK/ACC)	[252]
	In bovine VSMC *(sex not reported)*, it inhibits cell proliferation in the G1-S phase probably by inhibition of ERK1/2	[253]
	In caco cells *(sex not reported)*, it inhibits maltase, human sucrose, glucose transport across Caco-2 monolayers, and uptake of glucose by GLUT2 in Xenopus oocytes; it is a weak inhibitor of human *α*-amylase	[254]
	In vitro, it inhibits platelet aggregation being less active than HTyr. In whole blood, collagen platelet aggregation is not modified (*sex not reported*)	[209]
	It is antioxidant both in chemical assay and in the lag phase prolonging of LDL oxidation. However it is less active than homovanillic alcohol	[255]
	In samples of pooled human liver microsomes (*sex not reported*), it inhibits CYP3A	[256]
	J774A.1 cells (*sex not reported*) and in peritoneal macrophages from *male* mice, it increases the production of NO that is blocked by NOS inhibitor	[257]
	In *male* rat peritoneal leukocytes triggered by calcium ionophore, it inhibits 5-LOX and exerts antioxidant effects when leukocytes are stimulated by PMA	[157]
	In human HUVEC *(sex not reported)*, oleuropein and HTyr reduces the stimulated tube-like differentiation and stimulates locomotion, the increase in MMP-9 secretion induced by PMA without affecting tissue inhibitors of MMP, with this activity being mediated by pretranslation process. It inhibits PMA-stimulated COX2 activity and expression. HTyr before decreases intracellular ROS and nuclear translocation of the p65 NF-*κ*B and its transactivation	[216]
	In pooled human liver microsomes (*sex not reported*), they inhibit androstenedione 6*β*-hydroxylase and 17*β*-HSD	[210]
Oleuropein glycoside	In diluted human blood cultures (*sex not reported*) stimulated with LPS, it decreases IL-1*β*	[158]
Oleuropein, caffeic acid, Tyr HTyr	In acellular models, they scavenger reactive nitrogen species, with Tyr being the less active; however they do not inhibit the nitrergic transmission in the nerve-stimulated anococcygeus preparation of *male* rats	[258]
Oleuropein-containing supplement OPIACE	In DM2 model (Tsumura Suzuki obese diabetes *male*) mice, the diet attenuates hyperglycaemia and impairs glucose tolerance and oxidative stress but has no effect on obesity	[259]
Olive water methanol extract	In normotensive anaesthetized and atropinized rats (*sex not reported*), the intravenous administration of extract reduces the BP. In isolated atria of Guinea pig *of both sexes*, it reduces the spontaneous beating. In isolated thoracic artery of *male* and *female* rabbits it reduces K^+^ and/or phenylephrine induced contraction	[260]
Pinoresinol	Using DPHH test, it exerts antioxidant effects being more active than acetoxypinoresinol	[156]

	In PMA-stimulated RAW 264.7 macrophages (*sex not reported*), Tyr decreases the O_2_^−^ and H_2_O_*2*_ generation induced by PMA and scavenges the O_2_^−^. These effects seem to be linked with the impairment of (3H)AA release, COX2 expression, PGE_2_/B4 synthesis, and NO release	[261]
	In RAW 264.7 macrophages (*sex not reported*), triggered by oxLDL-stimulated Tyr reverts H_2_O_*2*_ generation and the AA release and PGE_2_ production	[262]
	In human monocytes (*sex not reported*) stimulated with PMA, it reduces the production of O_2_^−^ and the expression of mRNA and protein of COX2, dose-dependently decreasing PGE_2_ production	[220]
Tyr	In RAW 264.7 macrophages (*sex not reported*), it reduces the activation of iNOS and COX2 gene expression, NF-*κ*B, interferon regulatory factor-1 (IRF-1), and activator of transcription-1*α* (STAT-1*α*) induced by gliadin + IFN-*γ*	[263]
	In *male* rat peritoneal leukocytes triggered by calcium ionophore, it inhibits 5-LOX and exerts antioxidant effects when leukocytes are stimulated by PMA	[157]
	In human PBMC (*sex not reported)* and HL60 cells, it inhibits H_2_O_2_‐induced DNA damage	[185]
	In PBMC obtained by *healthy men and women*, it inhibits the increase of IL‐1*β*, MIF, and RANTES induced by oxysterols	[228]

Tyr, Tyr glucuronate (Tyr-GLU), and sulfate (Tyr-SUL)	In TNF-*α* treated-HUVEC *(sex not reported*), Tyr and Tyr-SUL prevent ROS generation and GSH decrease and downregulate GPx-1, GCL, and OH-1 genes. Tyr-SUL, Tyr, and Tyr-GLU prevent the phosphorylation of NF-*κ*B signaling proteins. Tyr-GLU and Tyr-SUL prevent the increase of genes and proteins expression and secretion of adhesion molecules. *In vivo*, Tyr and Tyr-SUL, in a dose-dependent manner, ameliorate plantar and ear edemas in *male mice*	[264]
Tyr, oleuropein, and olive pomace	In anoxic EA.hy926 human endothelial cell line *(sex not reported*), both Tyr and oleuropein attenuate anoxia-induced expression of MMP-9 and MMP-2. Tyr is more efficient than oleuropein in reducing TNF-*α*. The olive pomace ameliorates all the above parameters and induces time-dependent phosphorylation of p38 MAPK and ERK1/2, and inhibits anoxia-induced NF-*κ*B activation.	[265]
Verbascoside	In PBMC *(sex not reported)* and HL60 cells, it inhibits H_2_O_2_^−^ induced DNA damage.	[185]

**Table 3:** Clinical studies on the effect of EVOO, VOO, OO, leaf extracts, and MPCs.

Compounds	Individuals	Design	Main data	References
High MPC EVOO vs moderate and low MPC EVOO	200 healthy *men*	Multicenter RC crossover design	The negative association between the oleic/linoleic acid ratio and biomarkers of oxidative stress and improvement of LDL fatty acid profile	[296]
EVOO *vs* saturated fat diet	18 healthy postmenopausal *women*	Prospective, longitudinal, study	EVOO decreases the risk to develop the metabolic syndrome and CAD	[297]
EVOO *vs* soya oil	41 adult *women* with excess body fat	Double-blinded RC vs placebo	EVOO increases fat loss and reduces DBP and some biochemical parameters	[298]
High MPC EVOO *vs* low MPC EVOO	9 *men* and 11 *women* with metabolic syndrome	RC sequential crossover design	After EVOO-based breakfast, numerous inflammatory genes involved in factor NF-*κ*B, AP-1, MAPK, and AA pathways are repressed in PBMC	[299]
High MPC VOO vs intermediate and low VOO	19 *men* and 30 *women* with metabolic syndrome	RC, crossover design	High MPC VOO-based breakfast attenuates plasma LPS, TLR4, and SOCS3 proteins, activation of NF-*κ*B and the IL-6 vs low and intermediate oil. In PBMC, postprandial expression of IL-1B, IL-6, and CXCL1 is reduced especially by high MPC VOO	[300]
High MPC EVOO *vs* low MPC EVOO	*6 healthy men* and *6 healthy women; 6 men* and *6 women* with metabolic syndrome	Paired study	Acute high MPC EVOO transiently improves glycaemia and insulin sensitivity. It directly modifies the miRNA of PBMC. Acute EVOO poor in MPC is less effective	[278]
EVOO *vs* ROO	14 healthy and 14 hypertriacylglycerolemia *men*	Blind RC crossover design	EVOO has postprandial anti-inflammatory effects	[301]
EVOO	*26 male and 34 female* DM2 patients	RC trial	Both atorvastatin and EVOO reduce plasma lipids and increase HDL with a higher activity of atorvastatin	[302]
EVOO	17 *males* and 13 *females* with impaired fasting glucose	Blind RC crossover design	After EVOO meal, glucose, TG, ApoB-48, and DPP4 activity decrease, whereas insulin and GLP-1 increase *vs* meal without EVOO. Chol and HDL do not change after EVOO meal *vs* meal without EVOO	[303]
EVOO *vs* coconut oil *vs* unsalted butter	Healthy *women (67%)* and *men (33%)*	RC trial	No changes in BW, BMI, central adiposity, fasting blood glucose, SBP, and DBP for all diets. Butter increases LDL; coconut increases HDL	[304]
EVOO *vs* VOO	*41 males* and *females* (overweight or obese)	Single-blinded RC	EVOO decreases SBP and increases anti-CD3/anti-CD28 stimulated T cell proliferation *vs* VOO	[305]
VOO rich in MPC *vs* ROO	*11 women* at stage 1 of essential hypertension or 13 with normal-high BP	Double-blind RC crossover design	VOO rich in MPC decreases SBP, DBP, CRP, LDL, ADMA and increases nitrites/nitrates and hyperemic area after ischemia	[306]
Diet enriched with VOO, walnuts, or almonds	*9 female and 9 male* hypercholesterolemic patients	RC crossover design	The VOO, walnut, and almond diets reduce LDL; They reduce LDL, Chol, and LDL/HDL ratio. Other lipid fractions, oxidation, and inflammatory biomarkers do not change	[307]
OO rich in MPC *vs* OO + EGCG	Patients with endothelial dysfunction, OO rich in MPC (13 *men* and 15 *women*) OO + EGCG (10 *men* and 14 *women*)	Double-blinded RC	They reduce endothelial dysfunction, but only OO reduces inflammatory biomarkers, white blood cells, monocytes, and lymphocytes	[308]
OO enriched with oleanolic acid (OA) *vs* OO	176 individuals of *both sexes* with impaired fasting glucose and impaired glucose tolerance	Multicenter double‐blind RC trial	The intake of OO rich in OA reduces the risk of developing DM in individuals with impaired fasting glucose and impaired glucose tolerance	[309]
MedDiet + EVOO *vs* MedDiet + nut *vs* control	7447 old participants of PREDIMED *(43% men* and *57% women)* at risk for CVD	Observational study in primary prevention	Long intake of MedDiet + EVOO and MedDiet + nut reduces primary CV events	[11]
High MPC EVOO *vs* moderate and low MPC VOO	18 *healthy men*	Double-blind RC, crossover design	High PMC EVOO reduces SBP *vs* basal values and low PMC VOO. It maintains DBP values compared to low MPC VOO. Further, it reduces ACE and NR1H2 gene expressions *vs* basal and IL-8RA *vs* low PMC MPC	[310]
MeDiet + EVOO vs MeDiet + washed EVOO vs habitual diet	*26 healthy men* and *64 healthy women*	RC crossover design	In plasma, MedDiet + EVOO reduces oxidative and inflammatory status. In PBMC, it reduces oxidative stress, the gene expression of INF-*γ*, Rho GTPase-activating protein 15, IL-7 receptor, adrenergic *β*2 receptor and polymerase (DNA-directed) k. These effects with the exception of polymerase (DNA-directed) *κ* are more elevated when EVOO rich in polyphenols was added	[311]
High MPC EVOO *vs* low MPC EVOO	46 healthy subjects *(14 men* and *32 women)*	RC crossover design	No effect on fasting plasma lipids, oxLDL, and LPO	[106]
EVOO *vs* refined OO	24 *men*	RC crossover design	Only EVOO rich in MPCs lowers oxLDL being ineffective *vs* plasma lipids	[312]
High MPC VOO *vs* moderate and low MPC VOO	18 healthy *men*	RC crossover design	High MPC VOO reduces oxLDL MPC-1, CD40L, IL-23A, IL-7R, IL-8RA, ADRB2, and OLR1 genes, whereas IFNG, IL-7R, IL-23A, CD40L, MCP-1, and IL-8RA decrease with low MPC VOO	[313]
High MPC VOO + triterpenes (OVOO) *vs* OVOO + higher MPC and triterpenes (FOO) vs low MPC and triterpenes (VOO)	27 healthy *men* and 26 healthy *women*	Double-blind RC, crossover design	Urinary 8-hydroxy-2′-deoxyguanosine, plasma IL-8, and TNF- *α* decrease more after FOO vs OVOO	[314]
High MPC VOO + triterpenes (OVOO) *vs* OVOO + higher amounts of MPC and triterpenes (FOO) vs low MPC and triterpenes (VOO)	27 healthy *men* and 26 healthy *women*	Double-blind RC, crossover design	After OVOO, HDL increases only in females. Chol increases after FOO and TG after VOO and OVOO. SBP decreases after the VOO and increases after the FOO. DBP and pulse pressure do not vary as well as LDL, sICAM-1, and sVCAM-1. Plasma ET-1 decreases after the VOO, OVOO, and FOO	[315]
VOO, VOO + MPC (FVOO), VOO + MPC + Thyme phenols (FVOOT)	Hypercholesterolemic *men* and *women*	Double-blind RC crossover design	Acute and sustained intake of VOO and FVOO attenuate PON1 protein and increase PON1-associated specific activities, while FVOOT has opposite effects. Only VOO increases PON3 protein	[316]
VOO *vs* VOO + MPC (FVOO) vs VOO + MPC + Thyme phenols (FVOOT)	Hypercholesterolemic volunteers: 5 women *and 7 men*	Double-blind RC, crossover design	FVOOT reduces serum oxLDL and elevates gut bifidobacteria vs VOO. FVOO does not change blood lipids and microbial populations but elevates the coprostanone vs FVOOT	[317]
VOO *vs* VOO + MPC (FVOO) VOO + MPC + Thyme phenols (FVOOT)	Hypercholesterolemic volunteers:*19 men* and *14 women*	Double-blind, RC crossover design	Urinary HTyr sulfate and thymol sulfate increase after FVOO or after FVOOT, respectively. FVOO and FVOOT do not change glycaemia, TG, LDL, HDL, ApoAI, and ApoB100 *vs* VOO with the exception of LDL that decreases after FVOO. FVOO and FVOOT change the lipoprotein subclasses profile and decrease insulin resistance index. BP and BMI do not change	[318]
VOO *vs* VOO + MPC (FVOO)	Prehypertensive or stage 1 hypertension participants (7 *men* and 6 *women*)	Double-blind RC crossover design	FVOO decreases ischemic reactive hyperemia, oxLDL, postprandial glycaemia, TG, PAI-I, and CRP *vs* VOO	[319]
VOO *vs* VOO + MPC and VOO + Thyme	*8 men* and *14 women* hypercholesterolemic subjects	Double-blind, RC crossover design	In PBMC, the intake of enriched VOO and VOO + thyme increases the expression of proteins involved in Chol efflux and nuclear receptor-related genes	[320]
VOO *vs* VOO + MPC (FVOO) and VOO + Thyme (FVOOT)	Hypercholesterolemic subjects: *19 men* and *14 women*	Double-blind, RC crossover design	The 2 enriched oils elevate antioxidants in HDL, whereas *α*-tocopherol is elevated only after FVOOT	[321]
VOO *vs* VOO + MPC *vs* VOO + MPC + Thyme phenols	19 hypercholesterolemic *men* and 14 *women*	Double-blind RC crossover design	Their consumption of each oil affects the HDL proteome in a cardioprotective mode	[322]
Diets with VOO and refined OO vs sunflower or corn oil during washout period	24 young *women* with high-normal BP or stage 1 essential hypertension	Double-blind RC crossover design	Only VOO decreases SBP and DBP, serum asymmetric dimethylarginine, oxLDL, and CRP. It increases the plasma nitrites/nitrates ratio and hyperemic area after ischemia	[306]
High MPC OO enriched breakfast *vs* low MPC OO breakfast	5 hypercholesterolemic *men* and 16 *women*	RC design sequential crossover	After the high MPC breakfast, FVIIa increases less and PAI-1 activity decreases more than after the low MPC breakfast	[169]
OO rich in MPC *vs* refined OO	69 healthy participants of *both sexes*	Double-blind RC parallel design	Both OO improve the urinary proteomic CAD score but not chronic kidney disease or DM proteomic biomarkers. No differences are measured between the two OO	[99]
OO with high *vs* OO with moderate MPC	pre/hypertensive patients 17 *men* and *6 women*	RC crossover design	In white blood cells, high MPC OO increases gene expression of ATP binding cassette transporter-A1, scavenger receptor class B type 1, PPAR*α*, PPAR*γ*, PPAR δ, and CD36 *vs* moderate MPC OO	[323]
High MPC OO *vs* moderate MPC and low MPC OO	30 *healthy subjects of unknown sex*	Double-blind RC vs placebo- crossover design	The consumption of oil rich in MPCs increases MPCs in LDL-C and decreases oxLDL	[324]
High MPC OO *vs* moderate and low MPC OO	12 healthy *male* subjects	Double-blind RC, crossover design	All OO promote postprandial increase in F2-isoprostanes whereas the LDL oxidation is inversely linked with MPCs	[325]
High MPC OO *vs* moderate and low MPC OO	200 healthy *men*	RC crossover design	HDL and Chol increase and decrease linearly with the MPC amounts, respectively. OxLDL and MPC amount are inversely related. TG decrease is not influenced by MPC amount	[325]
High MPC OO *vs* low MPC OO	10 menopausal healthy *women*	RC design crossover	MPC-rich OO diet reduces DNA damage vs low MPC OO whereas plasma antioxidant capacity does not diverge	[326]
High MPC OO *vs* moderate and low MPC OO	12 *male* healthy subjects	Double-blind, RC crossover design	Short-term consumption of MPC-rich OO decreases plasma oxLDL, urinary 8-oxo-dg and increases plasma HDL and GPx *vs* moderate and low MPC OO	[327]
High MPC OO	Patients with polymorphism in NOS3 Glu298Asp (rs1799983) of eNOS (*22 men, 35 women*)	RC sequential crossover design	Single administration seems to reduce the deleterious effect of the T allele carrier's condition	[328]
High MPC OO vs moderate and low MPC OO	30 healthy men from a religious center	RC, crossover design	MPC-rich OO is more effective in protecting LDL oxidation and in raising HDL than OO with lower quantities of MPCs	[15]
High MPC OO *vs* low MPC OO	22 mildly dyslipidemic subjects	RC crossover design	MPC-rich OO lowers plasma TXB_2_ and elevates plasma antioxidant capacity *vs* low MPC OO. Urinary F2-isoprostanes and plasma lipids do not diverge between the two groups	[329]
High MPC OO *vs* low MPC OO enriched breakfast	21 hypercholesterolemic subjects *(5 men* and *16* postmenopausal women)	RC crossover design	High MPC OO protects against postprandial endothelial dysfunction and decreases lipid peroxide and F2-isoprostanes *vs* low MPC OO	[330]
High MPC OO vs low phenolic OO	28 individuals with CHD (*sex not reported)*	Double-blind RC placebo-controlled, crossover design	Enriched OO decreases IL-6 and CRP being ineffective on soluble sICAM-sVCAM-1 and lipid profile	[331]
High MPC OO vs low MPC OO *vs* corn oil	12 healthy men	The study has a Latin square design	Enriched OO decreases TXB2 and LTB4 and increases plasma antioxidant capacity	[332]
High MPC OO *vs* low MPC OO	40 *men* with stable CID	RC crossover design	MPC-rich OO decreases oxLDL and LPO and increases GPx	[333]
OO *vs* sunflower-seed *vs* and rapeseed	18 healthy *men*	Double-blind RC crossover design	Postprandial lipid and lipoprotein concentrations are not greatly affected versus rapeseed and sunflower-seed oil, while rapeseed and OO diets have the same effect on LDL oxidation	[334]
OO	18 healthy *men*	RC crossover design	OO may attenuate the acute procoagulant effects of fatty meals	[335]
OO	8 *men* and 5 *women* with type DM2	Single-blinded RC crossover design	It increases in GLP-1 and GIP	[336]
OO (unrefined)	23 hypertensive patients *of both sexes*	Double-blind RC crossover design	Resting SBP and DBP are significantly lower at the end of the MUFA diet *vs* the PUFA diet. The cold pressor test and isometric exercise are similar. Daily drug dosage is significantly reduced during the MUFA *vs* PUFA diet	[337]
High MPC OO *vs* low MPC OO	Healthy smokers: 11 *men* and 14 *women*	Single-blind RC crossover design	Plasma antioxidant capacity and oxLDL do not differ significantly between the rich and low MPC OO	[18]
High MPC OO (HPCOO); low MPC VOO low-MPCOO (LPCOO), refined OO	25 healthy *men*	RC parallel, crossover, design	HPCOO decreases ApoB-100 and small LDL particles vs baseline and LPCOO. LPCOO increases previous parameters. HPCOO increases the lag time of LDL oxidation, which is not affected by LPCCO. LPL gene expression is not significantly changed by both OO	[338]
High MPC OO (HPCOO); VOO low MPC OO (LPCOO); refined OO	47 healthy men	RC crossover design	HPCOO increases HDL cholesterol efflux capacity *vs* the LPCOO and incorporation of MPC and their metabolites in HDL and HDL2. HPCOO intake decreases HDL3 and the HDL core becomes TG-poor, and HDL fluidity increased	[339]
HTyr	Healthy subjects (*12 men* and *16 women)*	Double-blinded, RC crossover design	Regular intake of HTyr improves the antioxidant defense and decreases nitrate and MDA	[340]
HTyr	21 healthy volunteers (*sex not reported*)	Double-blinded, RC crossover design	In PBMC, it induces miR-193a-5p, which leads to the generation of anti-inflammatory molecules	[218]
Oleuropein	24 healthy participants (*sex not reported*)	Double-blind RC Latin square design	No effect on postprandial glucose derived from bread, but in solution it attenuates postprandial blood glucose after 25 g sucrose, but has no effect after 50 g of sucrose or glucose	[254]
Oleuropein	Healthy 10 *men* and *10 women*	Double-blind RC crossover study	Its intake lowers glycaemia, DPP‐4 activity, soluble NADPH oxidase‐derived peptide activity, 8‐iso‐PGF2*α*, platelet p47^phox^ phosphorylation and elevates insulin and GLP‐1	[341]
Low-fat diet *vs* high in saturated fat (butter) *vs* high in monounsaturated fat (EVOO) diets	8 *women* and 5 *men* with type 1 DM	RCT crossover design	The addition of EVOO attenuates the early postprandial glucose response	[342]
Lunch + EVOO	17 men and 13 women patients with impaired fasting glucose	RCT crossover design	Lunch + EVOO reduces glucose, TG, ApoB-48, and DPP4 activity and increases insulin and GLP1. Chol and HDL do not change	[303]
Lunch + EVOO	*12 healthy men* and *13 healthy women*	RC crossover design	Lunch + EVOO decreases postprandial glucose and LDL	[343]
Lunch + EVOO vs lunch + corn oil	Healthy subjects *(12 men and 13 women)*	RCT crossover design	Lunch + EVOO ameliorates postprandial oxidative stress and endothelial dysfunction being lunch + corn oil ineffective	[344]
Lunch + EVOO	30 patients with impaired fasting glucose	RC crossover design	Lunch+EVOO attenuates the increase of oxidative stress and in LPS	[345]
Lunch + EVOO	Subgroup of the PREDIMED study, *110 women* with metabolic syndrome	Multicenter, controlled parallel group	MedDiet + EVOO decreases urinary 8-oxo-7,8-dihydro-2′-deoxyguanosine and prostanoids	[346]
MedDiet + EVOO *vs* MedDiet + nuts *vs* MedDiet with advice to use low fat	7477 individuals (57% women) at high CV risk	Randomized multicenter PREDIMED study testing the MedDiet in primary CV prevention	MedDiet + EVOO and MedDiet + nuts reduce the incidence of major CV events by approximately 30% *vs* the control diet	[347]
MedDiet + EVOO *vs* MedDiet + nuts *vs* MedDiet with advice to use low fat	2292 (*1343* women) patients with high CV risk 2210 (*1200* women) 2203 (*1323* women)	Post hoc analysis of the PREDIMED study	MedDiet + EVOO reduces the risk of atrial fibrillation	[348]
MedDiet + EVOO *vs* MedDiet + nuts *vs* MedDiet with advice to use low fat	351 men and women with DM2 or CV risk ≥3	A subgroup of PREDIMED study	MedDiet + EVOO decreases the BW and changes fat distribution	[349]
MedDiet + EVOO *vs* MedDiet + nuts *vs* MedDiet with advice to use low fat	*Men* and *women* (3541 patients) at high CV risk	PREDIMED study	The MedDiet + EVOO reduces DM2 risk among persons with high CV risk	[350]
MedDiet + EVOO *vs* MedDiet + nuts *vs* MedDiet with advice to use low fat	3230 *men* and *women* with DM2	PREDIMED study	MedDiet + EVOO may delay the introduction of glucose-lowering medications	[351]
MedDiet + EVOO *vs* MedDiet + nuts, low-fat diet	Old *men* and *women*	PREDIMED study	MedDiet especially if supplemented with EVOO changes the transcriptomic response of genes related to CV risk	[352]
MedDiet + EVOO *vs* MedDiet + nuts, low-fat diet	Old *men* and *women*	PREDIMED study	Both diets decrease IL-6, IL-8, MCP-1, and MIP-1*β*. MedDiet + EVOO decreases IL-1*β*, IL-5, IL-7, IL-12p70, IL-18, TNF-*α*, IFN*γ*, GCSF, GM-CSF, ENA78, E-selectin, and sVCAM-1 *vs* the MedDiet + nuts group	[353]
MedDiet + EVOO *vs* MedDiet + nuts, low-fat diet	160 (*74 men* and *86 women*) with high CV risk	PREDIMED study subgroup	Both diets reduce CRP, IL-6, TNF-*α*, and MCP-1. After 3 years, both reduce CD49d and CD40 expressions in T lymphocytes and monocytes and increase HDL but decrease Chol, LDL, TG, and BP. At 5 y, low-fat diet increases glucose and glycated hemoglobin	[354]
MedDiet *vs* MedDiet + EVOO MedDiet + corn oil	12 *men* and 13 *women*	RC crossover design	EVOO but not corn oil counteracts the upregulation of NOX2 protecting from postprandial oxidative stress	[344]
MedDiet rich in OO	805 patients (*sex not reported*) with CHD, who had their last coronary event more than 6 months before enrolment, stratified in diabetes and prediabetes	Prospective, randomized, single-blind, controlled trial (CORDIOPREV)	MedDiet rich in OO improves endothelial function in patients with prediabetes and DM *vs* low-fat diet	[355]
Leaf extract	60 prehypertensive men	Double-blind, RC crossover design	It reduces plasma TC, LDL, TAG, HDL, Chol/HDL ratio, IL-8. It does not affect oxLDL, CRP, adiponectin, ICAM-1, VCAM-1, P-selectin, E-selectin, IL-6, IL-10, IL-1*β*, TNF-*α*, fasting glucose, insulin, fructosamine or calculated HOMA-IR or QUICKI indices, nitrites. It reduces SBP and DBP	[356]
Leaf extract	*9 male* and *9 female* healthy volunteers	Double-blind, RC crossover design	It modulates positively vascular functions and IL-8 production	[357]
Leaf extract	46 participants *(sex not reported)*	Double-blinded RC, placebo-controlled trial	It improves insulin secretion and sensitivity and increases IL-6, IGFBP-1, and IGFBP-2. It does not affect IL-8, TNF-*α*, CRP, lipid profile, BP, body composition, carotid intima-media thickness, or liver function	[358]
Leaf extract	152 patients with stage-1 hypertension (85.4% and 87.6% *women* in OO and captopril groups, respectively)	Double-blind RC	Leaf extract and captopril reduce SBP and DBP in a similar manner. Only leaf extract reduces TG	[359]

## Abbreviations

ACC: Acetyl-CoA carboxylase
ACE: Angiotensin converting enzyme
PI3 kinase: Phosphatidylinositol 3-kinase/Akt
ADAMTS: A disintegrin and metalloproteinase with thrombospondin motifs (aggrecanase)
AMPK: AMP-activated protein kinase
AP-1: Activator protein-1
AR: Androgen receptor
ALT: Alanine aminotransferase
AST: Aspartate aminotransferase
Chol: Cholesterol
COX: Cyclo-oxygenase
CRP: C reactive protein
CVD: Cardiovascular disease
DPP4: Dipeptidyl-peptidase-4
DPHH: 1,1-Diphenyl-2-picrylhydrazyl radical
ERK: Extracellular regulated mitogen-activated protein kinase
EDHF: Endothelium-derived hyperpolarization factor
EFSA: The European Food Safety Authority
eNOS: Endothelial nitric oxide synthase
ET: Endothelin
ET-1: Endothelin receptor-1
EGFR: Epidermal growth factor receptor
EET: Epoxyeicosatrienoic acid
ERK, PI3K/Akt/FOXO3a: Phosphoinositide 3-kinase/Akt/Forkhead box O3
FAS: Fatty acid synthase
FPPS: Farnesyl diphosphate synthase
GCL: Glutamate-cysteine ligase
GIP: Glucose-dependent insulinotropic polypeptide
GLP-1: Glucagon-like peptide-1
GM-CSF: Granulocyte-macrophage-colony-stimulating factor
GPx-1: Glutathione peroxidase 1
17-beta-HSD: 17-beta-hydroxysteroid dehydrogenase
HEL60: Promyelocytic leukemia cells
HMEC-1: Human microvascular endothelial cell line
HIF-1*α*: Hypoxia-inducible factor-1
ICAM: Intercellular adhesion molecule-1
iNOS: Inducible nitric oxide synthase
IL: Interleukin
JNK: c-Jun N-terminal kinase
LPS: Lipopolysaccharide
LPL: Lipoprotein lipase
LTB4: Leukotriene B4
IRF-1: Interferon regulatory factor-1
MDA: Malondialdehyde
MIF: Macrophage migration inhibitory factor
MMP: Matrix metalloproteinases
MAPK: Mitogen-activated protein kinases
MCP-1: Monocyte chemoattractant protein
MIP-1*α*: Macrophage inflammatory protein-1*α*
MPC: Minor polar compound
MPO: Myeloperoxidase
EGFR: Epidermal growth factor receptor
miRNAs: Micro-ribonucleic acids
NADPH oxidase: Nicotinamide adenine dinucleotide phosphate oxidase
NEP: Neutral endopeptidase
NO: Nitrogen oxide
NF-*κ*B: Nuclear factor-kappa B
Nrf2: Nuclear factor E2-related factor 2
oxLDL: Oxidized low-density lipoprotein
OH-1: Heme oxygenase-1
PAI-I: Plasminogen activator inhibitor-1
PI3: Phosphatidylinositol 3-kinase/Akt
PMA: Phorbol myristate acetate
PGI_2_: Prostacyclin
PPAR: Peroxisome proliferator activated receptor
PPAR*γ* coactivator-1*α*: Peroxisome proliferator activated receptor coactivator *γ*-1*α*
ROS: Reactive oxygen species
mTOR: Mammalian target of rapamycin
TXA_2_: Thromboxane A_2_
TXB_2_: Thromboxane B_2_
TRPA1: Transient receptor potential cation channel subtype A1
SIRT: Sirtuin
SREBP-1c: Sterol regulatory element binding protein 1c
STZ: Streptomycin
TG: Triacylglycerol
VCAM-1: Vascular cell adhesion molecule-1
VEGF: Vascular endothelial growth factor
VSMC: Vascular smooth muscle cells
Akt: Protein kinase B
CBS: Cystathionine *β*-synthase
CD: Cluster of differentiation
CSE: Cystathionine *γ*-lyase
EGFR: Epidermal growth factor receptor
FMO3: Flavin containing monooxygenase 3
p-Akt: Phosphorylated Akt
p-ERK: Phosphorylated.
